# Endoscopic Glucocorticoid Injection for the Treatment of a Refractory Benign Esophageal Stenosis in a Patient With Plummer-Vinson Syndrome

**DOI:** 10.7759/cureus.41896

**Published:** 2023-07-14

**Authors:** Kegan Jessamy, Amy Jessamy, Obiajulu Anozie

**Affiliations:** 1 Gastroenterology, Tidelands Health Gastroenterology, Georgetown, USA; 2 Medicine, The University of the West Indies, St. Augustine, TTO; 3 Critical Care Medicine, Northeast Georgia Medical Center Gainesville, Gainesville, USA

**Keywords:** endoscopic dilation therapy, triamcinolone acetonide, plummer-vinson syndrome, esophageal stenosis, gastroenterology and endoscopy

## Abstract

Plummer-Vinson syndrome (PVS) or Paterson-Brown-Kelly syndrome is a rare clinical condition characterized by the triad of esophageal webs/stenoses, iron-deficiency anemia, and progressively worsening dysphagia. It occurs mostly in Caucasian women in the fourth to seventh decades, particularly in northern countries. Esophageal webs and stenoses can be encountered during endoscopic evaluation for the patient’s complaint of dysphagia. Esophageal stenoses are characterized as simple or complex. A stenosis should be considered refractory once the patient has undergone several sequential dilatations within short intervals, optimized treatment for potential underlying causes (eosinophilic esophagitis or acid reflux), and after neuromuscular causes have been excluded. Glucocorticoid injection into a stenosis during an endoscopic dilation session has been proven to be beneficial as the initial treatment modality of refractory nonmalignant esophageal stenoses. We present a case of a 39-year-old woman with refractory esophageal stenosis in the setting of PVS which was successfully treated with serial endoscopic glucocorticoid injections while she received oral iron supplementation. To our knowledge, there are no previous cases of esophageal stenoses associated with PVS in the literature requiring endoscopic glucocorticoid injection for successful resolution.

## Introduction

Plummer-Vinson syndrome (PVS) or Paterson-Brown-Kelly syndrome is a rare clinical condition characterized by the triad of esophageal webs/stenoses, iron-deficiency anemia, and progressively worsening dysphagia [1]. When the syndrome occurs with iron deficiency in the absence of anemia, it is known as sideropenic dysphagia [2]. It occurs mostly in Caucasian women in the fourth to seventh decades, particularly in northern countries. Due to improvements in the nutritional status and lower rates of iron deficiency in this population, there has been a reduction in the prevalence of PVS [3]. Esophageal webs and stenoses can be encountered during endoscopic evaluation for a patient’s complaint of dysphagia. Esophageal stenoses are characterized as simple or complex. Simple stenoses are straight, concentric, and short (less than 2 cm in length) and allow the passage of a normal-diameter endoscope which is approximately 10 mm. Complex stenoses are irregular, angulated, longer (2 cm or greater in length), or have a severely narrowed diameter (less than 10 mm) [4]. We present the case of a 39-year-old woman with a refractory esophageal stenosis in the setting of PVS which was successfully treated with endoscopic glucocorticoid injections while she received oral iron supplementation.

## Case presentation

A 39-year-old woman presented to our clinic complaining of weight loss of 3.2 kg associated with dysphagia for three months. Her past medical history was significant for hypothyroidism, chronic menorrhagia, and gastroesophageal reflux disease. She had a surgical history of laparoscopic cholecystectomy and a prior cesarean section. She reported progressively worsening dysphagia to solid foods and pills with chronic heavy menstrual periods. She was referred to a gynecologist due to chronic menorrhagia and was treated with intramuscular medroxyprogesterone acetate every 12 weeks which stopped the episodes of menorrhagia. Due to pill dysphagia, she was started on ferrous sulfate solution 220 mg orally twice daily and she remained adherent with iron supplementation throughout her entire clinical course. Her weight was 47.6 kg (baseline 55-60 kg). The complete blood count and iron panel were obtained before and one month after oral iron supplementation (Table 1). Additional lab testing for weight loss showed a thyroid-stimulating hormone level of 1.150 μIU/mL and negative HIV and hepatitis B and C serology.

**Table 1:** Effect of oral iron supplementation on the complete blood count and iron panel.

	Before oral iron supplementation	One month after oral iron supplementation
Hemoglobin (g/dL)	7.4	8.2
Hematocrit	29.6%	32.4
Mean corpuscular volume (fL)	63.1	65.9
Red cell distribution width	24.2%	26.5
Eosinophils	1.0%	1.7%
Platelets (per μL)	334,000	367,000
Serum iron (μg/dL)	19	155
Total iron binding capacity (μg/dL)	486	444
Iron saturation	4%	35%
Ferritin (ng/mL)	3.5	4.5

**Figure 1:**
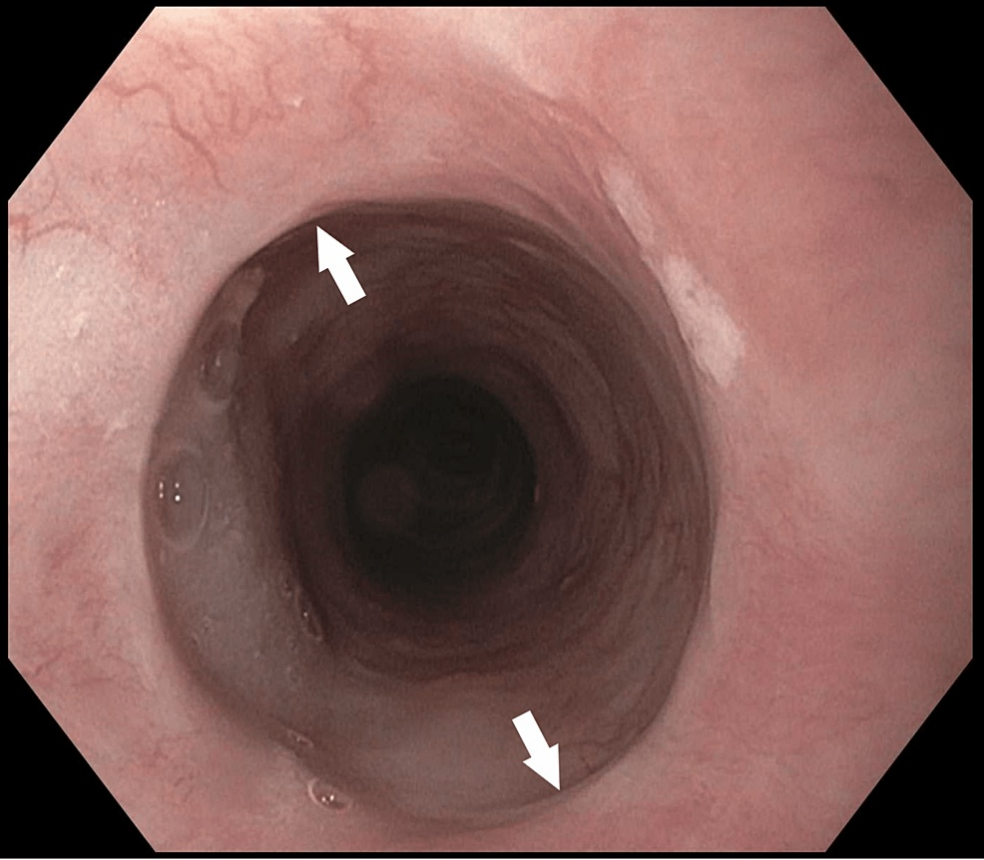
Esophageal web before endoscopic dilation (arrows).

A prior esophagogastroduodenoscopy (EGD) performed four years ago for dysphagia was normal. A repeat EGD was performed where she was found to have a benign-appearing 8 mm stenosis due to an upper esophageal web which was located 15 cm from the incisors (Figure 1), and normal mucosal appearance and normal luminal diameter in the middle and lower third of the esophagus.

**Figure 2:**
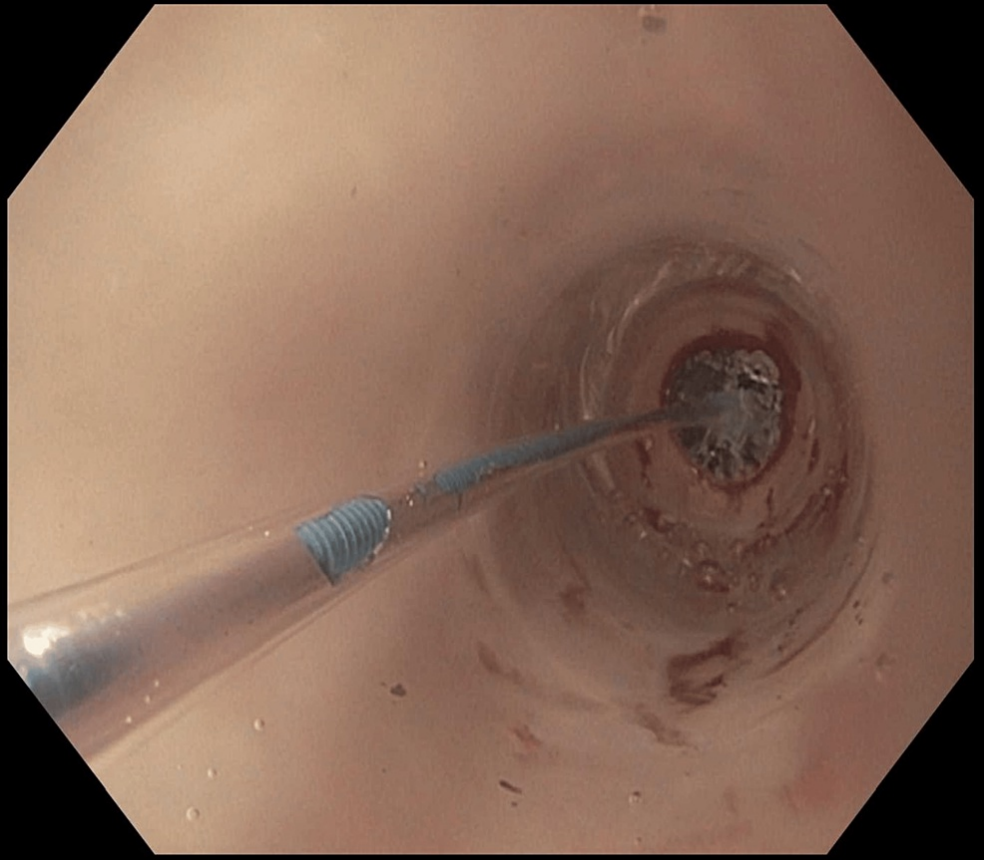
Through-the-scope balloon dilation with controlled radial expansion.

The upper esophageal stenosis at 15 cm from the incisors was dilated to 10 mm with a through-the-scope (TTS) balloon dilator. Biopsies of normal-appearing esophageal mucosa in the upper and lower third of the esophagus were negative for eosinophilic esophagitis (EoE) showing 1-2 eosinophils per high-power field (15 or more eosinophils per high-power field is diagnostic of EoE). She had a total of three sessions of endoscopic dilation in two to three-week intervals without improvement of the stenosis beyond 10 mm. On her fourth EGD, the upper esophageal stenosis was dilated to 15 mm with a TTS balloon (Figure 2), and 1 mL of triamcinolone acetonide (10 mg/mL diluted solution) was injected into each quadrant of the disrupted mucosa (total of 40 mg injected).

**Figure 3:**
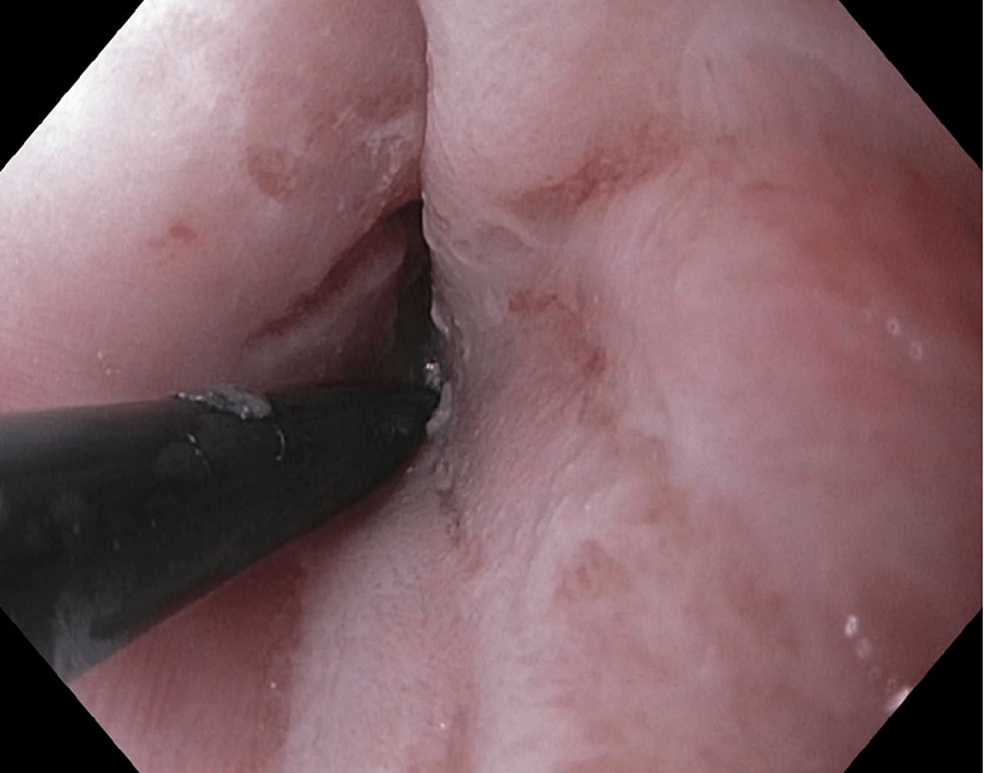
Glucocorticoid (triamcinolone) injection into disrupted esophageal mucosa following esophageal balloon dilation.

**Figure 4:**
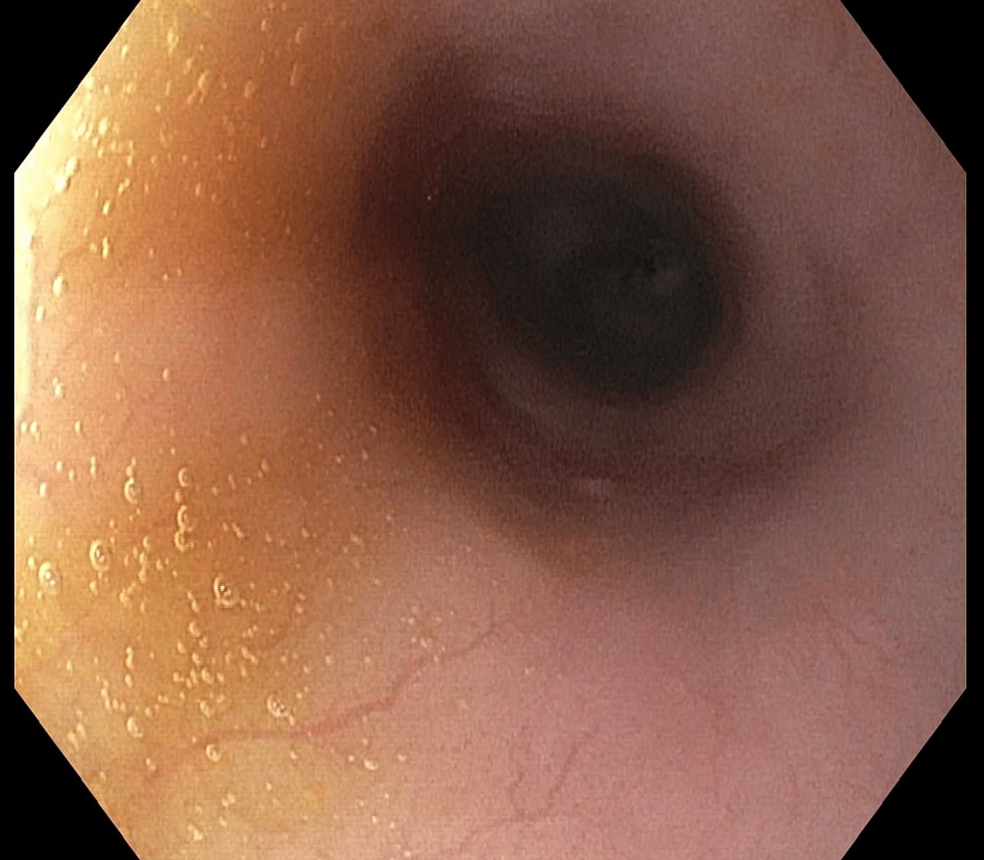
Site of the previous esophageal web following serial endoscopic dilations and two glucocorticoid (triamcinolone) injections.

Two weeks later, she had one additional session with TTS balloon dilation utilizing endoscopic glucocorticoid injection of triamcinolone (Figure 3), followed by three additional progressive dilations to 20 mm (Figure 4).

Each successive dilation was done in two to three-week intervals. She was adherent with omeprazole 40 mg orally daily (30-60 minutes before breakfast) during all esophageal dilations. With each endoscopic dilation, she reported improvement in her symptoms and eventually returned to a regular diet. Wireless pH monitoring over a 48-hour period (off of acid-suppressive medication for seven days) was done after the patient declined catheter-based pH monitoring and was negative for pathologic acid reflux (total acid exposure time 0.7% and DeMeester score 3.0). There was no gastrointestinal cause for anemia found on subsequent colonoscopy and small bowel video capsule endoscopy. Following medical therapy with iron supplementation and endoscopic therapy with triamcinolone and progressive TTS balloon dilations, she was found to have a weight of 64 kg (an increase of 20.9 kg from nadir).

## Discussion

Esophageal stenosis should be considered refractory once a patient has undergone several sequential dilatations within short intervals, optimized treatment for potential underlying causes (eosinophilic esophagitis or acid reflux), and after neuromuscular causes have been excluded [4]. Nonmalignant esophageal strictures with a length of less than 8 cm encountered during endoscopy can be effectively treated with balloon dilation with the pre-dilation diameter and stricture length influencing the number of repeat dilations [5]. The majority of esophageal stenoses can be successfully dilated to a diameter of 14 mm with less than five endoscopic sessions with about 10% having persistent dysphagia [4,6].

There is a lower risk of stenosis recurrence and fewer sessions of serial endoscopic dilation associated with endoscopic glucocorticoid injection. A meta-analysis (the majority of trials were nonblinded) involving 176 patients with refractory esophageal stenoses, glucocorticoid injection plus endoscopic dilation resulted in a lower risk of stenotic recurrence (relative risk = 0.64, 95% confidence interval (CI) = 0.51-0.81) and fewer endoscopic dilation sessions compared with dilation alone (mean difference = -1.06, 95% CI = -1.80 to -0.31) [7].

In the iron-deficient state in PVS, the gastrointestinal tract is susceptible and rapidly loses iron-dependent enzymes due to a high cell turnover, which leads to mucosal degeneration and web formation. Alternative proposed etiologies include genetic predisposition, malnutrition, and autoimmune processes [8]. Following esophageal dilation with mucosal disruption, there is local inflammation. Chronic inflammation leads to collagen deposition through the synthesis and the activation of multiple factors including transforming growth factor beta and alpha-2-macroglobulin (inhibitors of collagenase activity) [9]. Local glucocorticoid injection is thought to be a useful method to inhibit these inflammatory pathways. Four quadrant injection of a glucocorticoid at the location of stenosis during esophageal dilatation thus provides an appealing mechanism to decrease fibrosis and collagen deposition associated with chronic inflammation and minimize the likelihood of stenosis recurrence [4]. Glucocorticoid injection into stenosis during an endoscopic dilation session has been proven to be beneficial as the initial treatment modality of refractory nonmalignant esophageal stenoses [10,11].

It is unclear if the etiology of the stenosis influences the effect of steroid injection [4]. The usefulness of endoscopic glucocorticoid injection has also been proven in the prevention of benign esophageal stenoses occurring as a result of endoscopic submucosal dissection (ESD). Immediately following ESD, triamcinolone injection (100 mg) into the post-ESD ulcer reduces the rate of stenosis to 10-45% in cases of non-circumferential resection [12]. However, even after glucocorticoid injection, refractory stenoses requiring at least three sessions of endoscopic balloon dilatation developed in 19% of patients [12]. A randomized blinded trial involving 65 patients with postsurgical anastomotic stenoses evaluated triamcinolone injection (50 mg) directly into post-dilation disrupted mucosa and demonstrated that patients receiving triamcinolone required a fewer number of dilations to resolve stenoses (2.0 versus 4.0) and a greater number of patients did not have dysphagia at six months (39% versus 16%), these results are highly suggestive that injection into the disrupted mucosa may result in a greater effect on stenosis recurrence [13]. Some authors recommend that a maximum of three sessions of endoscopic steroid injections is appropriate [14,15].

Our success with regard to treating benign esophageal stenosis in our patient highlights the importance of considering endoscopic glucocorticoid injection as an initial treatment for refractory benign esophageal stenoses.

## Conclusions

To our knowledge, there have not been any previous cases of esophageal stenoses associated with PVS in the literature requiring endoscopic glucocorticoid injection for successful resolution. This case also shows the safety and feasibility of performing endoscopic triamcinolone injections for nonmalignant esophageal strictures, especially in the community-based hospital setting where access to metal, plastic, or biodegradable esophageal stents may be limited.
